# The mechanistic role of the thromboxane A2 receptor (TBXA2R) in non-small cell lung cancer (NSCLC)

**DOI:** 10.1186/s12935-026-04283-6

**Published:** 2026-04-28

**Authors:** Qiushi Wang, Asad Khan, Tianshun Zhang

**Affiliations:** The Hormel Institute, University of Minnesota, 801 16th Ave NE, Austin, MN 55912 USA

**Keywords:** TBXA2R, NSCLC, EGFR, NF-κB, Urethane-induced lung cancer

## Abstract

Non-small cell lung cancer (NSCLC) remains a leading cause of cancer-related death worldwide, underscoring the urgent need for novel therapeutic strategies. The thromboxane A2 receptor (TBXA2R) has gained attention as a pivotal molecule in cancer biology. Our findings reveal a marked overexpression of TBXA2R in NSCLC tissues compared with normal lung tissues, suggesting a possible association with tumorigenesis and disease progression. Furthermore, based on publicly available datasets, higher TBXA2R expression is associated with a reduced survival probability in NSCLC patients. Silencing TBXA2R expression significantly inhibits NSCLC cell proliferation, as evidenced by anchorage-independent growth assays and 3D culture models. RNA sequencing analysis revealed that TBXA2R contributes to NSCLC growth through the activation of the PI3K-AKT and cytokine-cytokine receptor interaction pathways. Notably, TBXA2R knockdown is associated with reduced EGFR expression, accompanied by changes in NF-κB p52 nuclear translocation and its subsequent binding to the EGFR promoter. Additionally, genetic ablation of TBXA2R in mice diminishes both the number and size of urethane-induced lung tumors. Collectively, this study suggests that TBXA2R is involved in NSCLC pathogenesis and may represent a potential therapeutic target. Inhibiting TBXA2R may improve NSCLC treatment outcomes and lessen the impact of this deadly disease.

**Supplementary Information:**

The online version contains supplementary material available at 10.1186/s12935-026-04283-6.

## Introduction

Non-small cell lung cancer (NSCLC), comprising adenocarcinoma, squamous cell carcinoma, and large cell carcinoma, constitutes around 85% of all lung cancer cases and remains a major contributor to cancer-related deaths globally [1–3]. The molecular complexity of NSCLC, characterized by various genetic mutations and signaling pathways, fuels both tumor development and therapeutic resistance [4, 5]. The aggressive nature of NSCLC, coupled with its resistance to conventional therapies, highlights the critical need to unravel its underlying pathophysiological mechanisms. Identifying biomolecules as viable therapeutic targets is crucial for advancing clinical outcomes of NSCLC [6, 7].

Recent studies have highlighted the role of TBXA2R and its pathways in the promotion and progression of various cancers. Dysregulated expression and activation of TBXA2R are implicated in tumor progression through several processes, such as enhancing cell proliferation, angiogenesis, invasion, and metastasis [8–15]. Thus, targeting the TBXA2R-mediated signaling pathways holds potential as a novel therapeutic approach, particularly for personalized treatments based on the patient’s TBXA2R expression profile.

Among the key genetic alterations in NSCLC, mutations in the *epidermal growth factor receptor (EGFR)* gene play a pivotal role. EGFR, a membrane-bound tyrosine kinase receptor, activates numerous downstream signaling pathways upon ligand binding and promotes cell growth, survival, and differentiation [16–18]. One of the primary pathways triggered by EGFR is the PI3K-AKT pathway, an essential signaling route that regulates cell survival, proliferation, and metabolism [19–22]. Additionally, EGFR signaling can activate the nuclear factor kappa B (NF-κB) pathway [23]. NF-κB is a transcription factor responsible for controlling the expression of genes associated with inflammation, immune responses, and cell survival [24]. EGFR activation leads to NF-κB activation through various intermediaries, resulting in its translocation to the nucleus and the subsequent promotion of genes involved in cytokine responses and tumorigenesis [24–26].

In this study, we investigated the clinical relevance of TBXA2R in NSCLC. We explored TBXA2R’s role in NSCLC progression and dissected the molecular mechanisms underlying its function. Furthermore, using a TBXA2R knockout mouse model, we assessed the impact of TBXA2R depletion on lung tumor development. These insights may contribute to more effective treatment strategies for this highly lethal disease.

## Materials and methods

### Reagents and antibodies

For cell culture, essential materials such as media, gentamicin, penicillin, and L-glutamine were purchased from Invitrogen, while non-essential amino acids were acquired from Corning (Corning, NY). Insulin was obtained from Gibco (Gaithersburg, MD), and fetal bovine serum (FBS) was sourced from Gemini Bio-Products (West Sacramento, CA). Laboratory reagents including Tris, NaCl, sodium bicarbonate, hydrocortisone, glucose, transferrin, epidermal growth factor (EGF), and SDS were purchased from Sigma-Aldrich (St. Louis, MO) for use in molecular biology assays and buffer preparation. Antibodies used in the experiments included the β-actin antibody (sc-47778) from Santa Cruz Biotechnology. The antibodies for p-PI3K (#4228), PI3K (#5405), p-AKT (#9271), AKT (#9272), p-mTOR (#5536), mTOR (#2972), NF-κB (p65) (#8242), and NF-κB (p52) (#37359) were all purchased from Cell Signaling Technology (Danvers, MA). The NF-κB (p50) polyclonal antibody (2430534) was purchased from Invitrogen, and the TBXA2R (TP receptor) polyclonal antibody (10004452) was acquired from Cayman Chemical (Ann Arbor, MI). For Western blot analysis, TrueBlot Ultra antibodies were used, and secondary antibodies, including anti-rabbit Ig HRP (18-8816-31) and anti-mouse Ig HRP (18-8817-31), were purchased from Rockland (Rockland, ME). These reagents were critical for the successful completion of the molecular and cellular experiments in this study.

### Cell culture and transfection

All cell lines, obtained from the American Type Culture Collection (ATCC), were cultured following ATCC protocols in a humidified incubator at 37 °C with 5% CO_2_. To ensure consistency across experiments, frozen vials of each cell line were used for up to 8 weeks of culture. NL-20 cells, an immortalized bronchial epithelial line, were maintained in Ham’s F12 medium, supplemented with 4% fetal bovine serum (FBS), 15,000 U penicillin, 15,000 U streptomycin, 2 mmol/L L-glutamine, 0.1 mmol/L nonessential amino acids, 10 ng/mL human recombinant epidermal growth factor (EGF), 0.005 mg/mL insulin, 500 ng/mL hydrocortisone, and 0.001 mg/mL transferrin. A549 human lung cancer cells were cultured in F-12 K medium containing 10% FBS and 1% antibiotics, while other human lung cancer cell lines were grown in RPMI-1640 medium supplemented with 10% FBS and 1% antibiotics.

### Lentiviral infection

Lentiviral plasmids were designed to target TBXA2R (shTBXA2R, #1: RHS3979-201745752, Clone Id: TRCN0000014174; #2: RHS3979-201745753, Clone Id: TRCN0000014175; #3: RHS3979-201745754, Clone Id: TRCN0000014176) and were purchased from Horizon Discovery (Cambridge, MA). Additionally, the pLKO.1-puro non-target shRNA control plasmid (shNT) was purchased from Sigma-Aldrich Co. LLC (St. Louis, MO). To facilitate TBXA2R knockdown, HEK293T cells were transfected using either the TBXA2R-targeting lentiviral vectors (shTBXA2R) or the shNT control, along with the packaging vectors pMD2.0G and psPAX. The transfection was performed with iMfectin Poly DNA transfection reagent (GenDEPOT, Barker, TX), following the recommended protocols. The transfection mixture, which was prepared in 10% FBS/DMEM without antibiotics, was added to the cells and incubated for 12 h. After this period, 10 mL of fresh medium containing antibiotics (penicillin and streptomycin) were introduced. Viral supernatant fractions were collected after 48 h, filtered through a 0.45-µm syringe filter, and utilized to infect the designated cells, supplemented with 8 µg/mL polybrene (Millipore). Following an overnight incubation, the medium was replaced with fresh complete growth medium containing the appropriate concentration of puromycin, and then cells were incubated for an additional 24 h. The resulting puromycin-selected cells were used in subsequent experiments.

### Western blot analysis

Western blotting was conducted following established protocols [27]. Primary antibodies were diluted 1:1000 and incubated overnight at 4 °C, followed by treatment with an HRP-conjugated secondary antibody diluted 1:5000. Protein bands were detected using a chemiluminescent substrate (GE Healthcare Biosciences, Piscataway, NJ).

### Anchorage-independent cell growth assay

To assess anchorage-independent cell growth, shCon and shTBXA2R cells (8 × 10^3^ cells/well) were suspended in a mixture of 1 mL Basal Medium Eagle (BME) and 0.3% Basal Medium Eagle agar supplemented with 10% FBS. This cell-agar mixture was then plated on 3 mL of solidified BME agar containing 10% FBS and 0.5% agar. After 2 weeks incubation, colonies were scored under a microscope by using the Image-Pro PLUS (v6.) software program (Media Cybernetics, Rockville, MD). Four photos were taken from each well and the colony numbers were counted. Each assessment was repeated 3 times, and the average number of colonies was compared between shNT- and shTBXA2R- expressing cells.

### 3-D cell culture

Vitrogel Hydrogel Matrix (VHM01) from The Well Bioscience (North Brunswick, NJ) was used for 3-D cell culture following the manufacturer’s instructions. Briefly, H1650 and H441 cells were first suspended in culture media. A total of 1 mL of Vitrogel was combined with 500 µL of the cell suspension. The hydrogel-cell mixture (300 µL) was dispensed into a 24-well plate at a density of 1 × 10⁵ cells per well, followed by a 15-min gelation at room temperature. Afterwards, 300 µL of medium was gently added to cover the hydrogel. Cultures were maintained at 37 °C with medium changes every 48 h. After 7 days, cells were collected for immunofluorescence analysis.

### Immunofluorescence analysis of 3-D spheroids and 2-D monolayers in H1650 and H441 cells

For 3-D culture, the cover medium was first removed from the hydrogel surface. PBS (100 µL) was then added and allowed to sit on the hydrogel for 1 min before being discarded. This wash step was repeated 3 times. Following this, 100 µL of 4% formaldehyde solution was applied to fix the spheroids for 30 min at room temperature. After fixation, the hydrogel was washed again 3 times with PBS. To permeabilize the cells, 100 µL of 0.1% Triton X-100 was added for 5 min at room temperature, followed by 3 additional PBS washes. The hydrogel was then blocked with 3% BSA in PBS for 60 min. Subsequently, the primary TBXA2R antibody was added directly to the blocking solution and incubated overnight at 4 °C. After overnight incubation, the hydrogel was washed 3 times with PBS, and then DAPI was added to stain the nuclei for 5 min in the dark. After incubation, the samples were ready for imaging under a fluorescence microscope.

For the 2-D monolayer culture, H441 or H1650 cells stably expressing shCon or shTBXA2R (4 × 10⁴ cells per well) were seeded onto 4-chamber slides. After 24 h, the cells were treated with urethane (4 µM) for 24 h. Following treatment, the cells were washed with PBS, fixed in methanol for 20 min, and rinsed twice with PBST (PBS containing 0.05% Tween-20). Blocking was performed using BSA/PBS (10 mg/mL) for 30 min, after which the cells were incubated overnight at 4 °C with primary antibodies, including those to detect NF-κB (1:200) or α-tubulin (1:500). After washing with PBST, the cells were incubated with 2% goat serum in BSA/PBS for 20 min. Secondary antibodies, Alexa Fluor 488 rabbit IgG or Alexa Fluor 594 mouse IgG conjugates, were then applied for 45 min. Finally, the cells were stained with Fluoro-gel II containing DAPI from Electron Microscopy Sciences (Hatfield, PA) for 30 min at room temperature. Samples were imaged using a Zeiss Axioskop microscope system.

### RNA extraction, library preparation, sequencing, and bioinformatics analysis

Total RNA was isolated using the RNeasy Mini QIAcube Kit (QIAGEN, Germantown, MD) according to the manufacturer’s guidelines, and RNA purity was assessed with the Bioanalyzer 2100 and RNA 6000 Nano LabChip Kit (Agilent, CA). Following RNA isolation and purification, mRNA was further enriched using Dynabeads Oligo (dT) (Thermo Fisher, Carlsbad, CA). The purified mRNA was then fragmented using the Magnesium RNA Fragmentation Module (New England Biolabs [NEB], Ipswich, MA, Cat. e6150). cDNA synthesis was conducted from these RNA fragments with SuperScript™ II Reverse Transcriptase (Invitrogen, Cat. 1896649). Dual-index adapters were ligated to the cDNA fragments, followed by size selection using Amperex beads. After treatment of U-labeled second-strand DNAs with a heat-labile UDG enzyme (NEB, Cat. m0280), the products were amplified by PCR. Sequencing was conducted using 2 × 150 bp paired-end reads (PE150) on an Illumina Novaseq™ 6000, adhering to the manufacturer’s protocol. RNA sequencing was performed by LC Science (Houston, TX).

For bioinformatics analysis, detailed methods are provided in the Supplementary Materials. The R package Limma (version 3.40.6) was used for differential expression analysis, comparing gene expression between the treatment and control groups. Genes were considered differentially expressed when the *p*-value was below 0.05 and the absolute fold change was ≥ 2. Enrichment analysis, including KEGG pathway analysis, was conducted using the NIH DAVID Bioinformatics Resource (https://david.ncifcrf.gov/tools.jsp).

### Quantitative real-time PCR

Total RNA was isolated using the RNeasy Mini QIAcube Kit (QIAGEN, Germantown, MD). To analyze gene expression for *TBXA2R*,* PCK1*,* LPAR4*,* CXCL14*,* INHBC*, and *EGFR*, 1 µg of total RNA per sample was used. Primer sequences for amplification are listed in Supplementary Table 1. Quantitative one-step real-time PCR was conducted using the TaqMan RNA-to-CT 1-Step Kit (Applied Biosystems, Foster City, CA), following the manufacturer’s guidelines. The expression levels of the target genes were normalized to glyceraldehyde 3-phosphate dehydrogenase (GAPDH) as a reference gene, ensuring uniform RNA input across samples.

### Luciferase reporter assay

H1650 or H441 cells (8 × 10⁴ cells per well) were plated in a 24-well dish and allowed to grow for 24 h. The cells were then transfected with an NF-κB luciferase reporter plasmid along with a β-galactosidase plasmid, serving as an internal control. Transfection was performed by using iMFectin poly DNA transfection reagent (GenDEPOT) as per the supplier’s protocol. Twelve hours post-transfection, the cells were exposed to 4 µM urethane for varying time periods (2, 4, 8, 16, or 24 h). Luciferase and β-galactosidase activity levels were determined using the Luminoskan Ascent and Multiskan MCC (Thermo Labsystems, Waltham, MA). Luciferase readings were adjusted based on the β-galactosidase activity to ensure accuracy.

### Chromatin immunoprecipitation (ChIP) assay

The binding motif of NF-κB on the *EGFR* promoter was predicted by using JASPAR (https://jaspar2022.genereg.net/downloads/) and MEME (https://meme-suite.org/meme/index.html). ChIP experiments were conducted using the Magna ChIP™ A/G kit from Millipore (Cat #17-10085). In brief, 1 × 10⁷ cells were fixed with 1% formaldehyde for 10 min, followed by quenching with 10× glycine for 5 min at room temperature. Cell lysates were sonicated to shear DNA to a size range of 200–1000 base pairs. Equal amounts of chromatin were then subjected to immunoprecipitation at 4 °C overnight using 10 µg of each of the following antibodies: NF-κB (p50), NF-κB (p52), NF-κB (p65), and rabbit IgG as a control. The immunoprecipitated complexes were collected with protein A/G magnetic beads, and qPCR analysis was performed using primers specific to the *EGFR* promoter.

### Animals and carcinogen treatment

Animal experiments were conducted following approval by the University of Minnesota Institutional Animal Care and Use Committee (Protocol ID: 1709–35106 A). TBXA2R mice were sourced from the Durham VA Medical Center (Durham, NC), while C57BL/6 mice were obtained from the Jackson Laboratory. Both wild-type (TBXA2R +/+) and knockout (TBXA2R -/-) mice were used in a urethane-induced lung cancer model. All mice were housed in virus- and antigen-free conditions, and standard PCR genotyping was conducted as per Jackson Laboratory protocols, using the following primers: fwd 5’-TGGGGGGTAGCTAGCTATGGTGTTC-3’, rvs 5’-GTGAGAAGGGCCGTGTGAT-3’, and ApoE NEO 5’-CTTCCTCGTGCTTTACGGTA-3’. At six weeks of age, the mice were assigned to one of four groups: WT receiving vehicle (4 males, 5 females), TBXA2R KO receiving vehicle (5 males, 5 females), WT treated with urethane (9 males, 9 females), and TBXA2R KO treated with urethane (10 males, 10 females). The urethane-treated groups were administered a single intraperitoneal injection of urethane (1 g/kg in PBS, Sigma) weekly for 10 weeks, while vehicle groups received PBS. Mice were monitored daily, weighed weekly, and euthanized by CO_2_ asphyxiation either at 30 weeks post-initial urethane injection or when moribund. Lung surface tumors were counted, and the lungs were collected for subsequent analyses.

### Immunohistochemical analysis of tissue array and mouse lung tissues

A human lung tissue array (BC041115d) was sourced from the cancer tissue bank collection at US Biomax Inc (Rockville, MD). Immunohistochemical staining procedures were performed using the Vectastain Elite ABC Kit (Vector Laboratories, Burlingame, CA), following the manufacturer’s guidelines. For mouse lung tissues, sections were embedded in paraffin, cut, and subjected to both hematoxylin and eosin (H&E) staining as well as immunohistochemical analysis. In brief, all tissue sections were deparaffinized and rehydrated. Antigen retrieval was achieved by boiling the sections in sodium citrate buffer (10 mM, pH 6.0) for 10 min. This was followed by a 10-min incubation in 3% hydrogen peroxide (H_2_O_2_) to block endogenous peroxidase activity. The sections were then blocked with 10% goat serum in PBS for 1 h at room temperature. Subsequently, the slides were incubated overnight at 4 °C with the primary TBXA2R antibody (1:100), while the mouse lung tissue sections were stained with an antibody against proliferating cell nuclear antigen (PCNA; 1:2000).

Following thorough washing, the sections were incubated for 1 h at room temperature with a secondary anti-rabbit antibody (1:150) from Vector Laboratories. The detection of staining was performed using the Vectastain Elite ABC Kit, with 3,3′-diaminobenzidine used as the chromogen. The sections were then counterstained with hematoxylin and visualized under a microscope at 200× magnification. Expression levels of target proteins were quantified using Image-Pro PLUS software (v.6, Media Cybernetics, Inc.), where integrated optical density (IOD) was measured to assess the staining intensity.

### Statistical analysis

Quantitative data are presented as mean values ± standard deviation (S.D.) or standard error (S.E.) from a minimum of 3 independent experiments or samples. To assess significant differences, either a student’s *t*-test or one-way ANOVA was used. A *p* value of less than 0.05 was considered indicative of statistical significance.

## Results

### Overexpression of TBXA2R in human lung adenocarcinoma is associated with patient survival probability

**Fig. 1:**
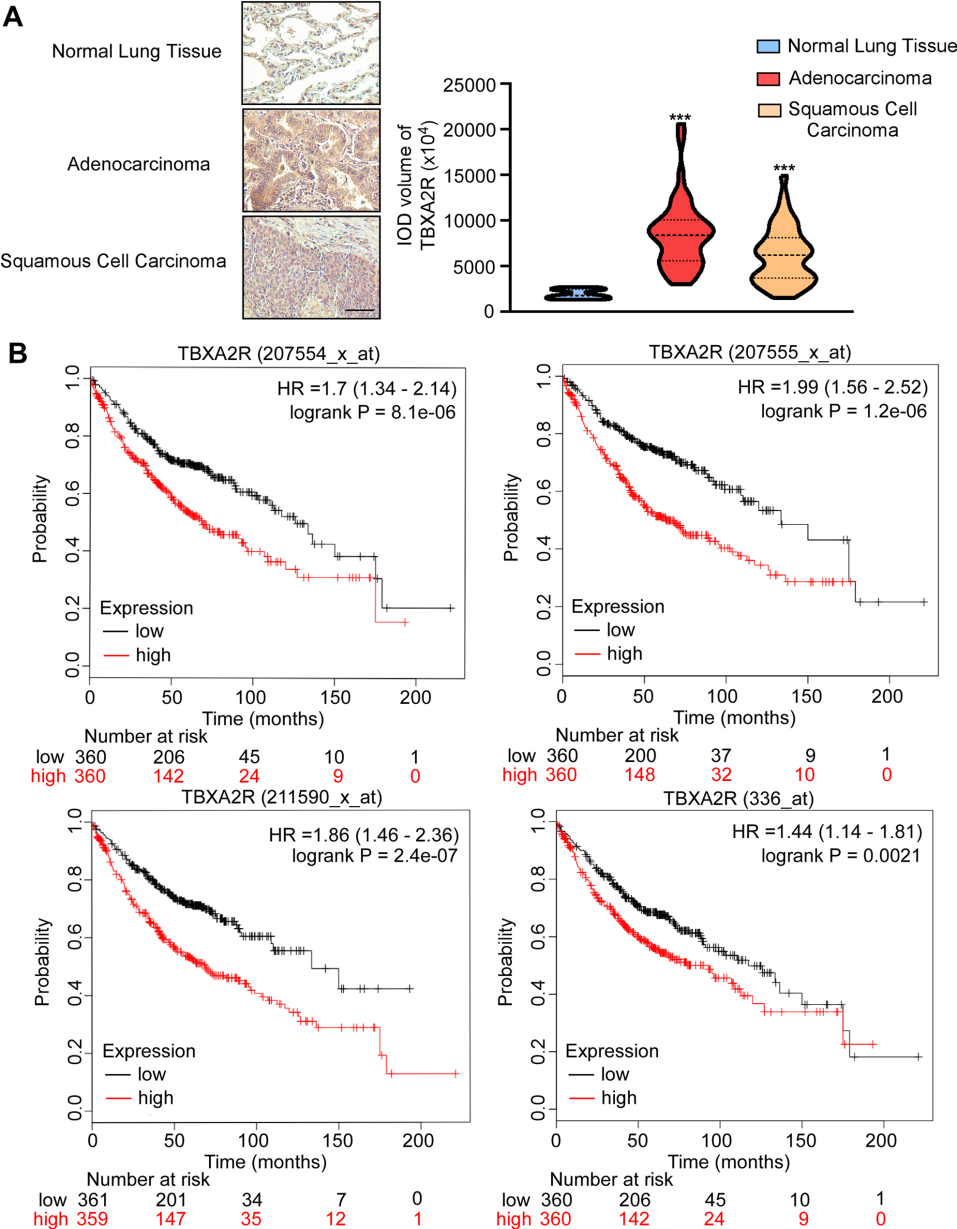
Elevated TBXA2R expression in human lung cancer and its impact on patient survival. **A**, immunohistochemical staining was performed to assess TBXA2R protein levels in both normal lung tissues and lung cancer specimens. TBXA2R was visualized using DAB (brown) staining, with hematoxylin used for counterstaining the nuclei (blue). Quantitative analysis of staining intensity was conducted, and statistical significance was evaluated using one-way ANOVA. The analysis included normal lung tissues (*n* = 10), adenocarcinoma tissues (*n* = 44), and squamous cell carcinoma tissues (*n* = 41); the scale bar denotes 100 μm. **B**, Kaplan-Meier survival analysis based on TBXA2R expression levels was conducted using the Kaplan-Meier Plotter tool (http://kmplot.com/analysis), incorporating Affymetrix probe IDs 207554_x_at, 207555_x_at, 211590_x_at, and 336_x_at for *TBXA2R*. A significant difference in TBXA2R expression between normal lung and adenocarcinoma tissues is indicated by asterisks (***, *p* < 0.001, one-way ANOVA)

Lung adenocarcinoma represents the most common type of primary lung cancer. Our examination of a lung cancer tissue array demonstrated that TBXA2R expression is increased in both adenocarcinoma and squamous cell carcinoma, with particularly pronounced expression observed in lung adenocarcinoma (Fig. 1A). To further explore the relationship between high TBXA2R expression and patient survival, we utilized the Kaplan-Meier plotter (https://kmplot.com/analysis/) for analysis (Fig. 1B). The results revealed that patients exhibiting elevated TBXA2R levels have a significantly reduced probability of survival compared to those with lower TBXA2R expression. In conclusion, our results indicate that TBXA2R is highly expressed in lung adenocarcinoma and correlates with poorer survival outcomes, underscoring its potential as a prognostic marker and a target for therapeutic strategies.

### Knockdown of TBXA2R reduces lung cancer cell growth

**Fig. 2:**
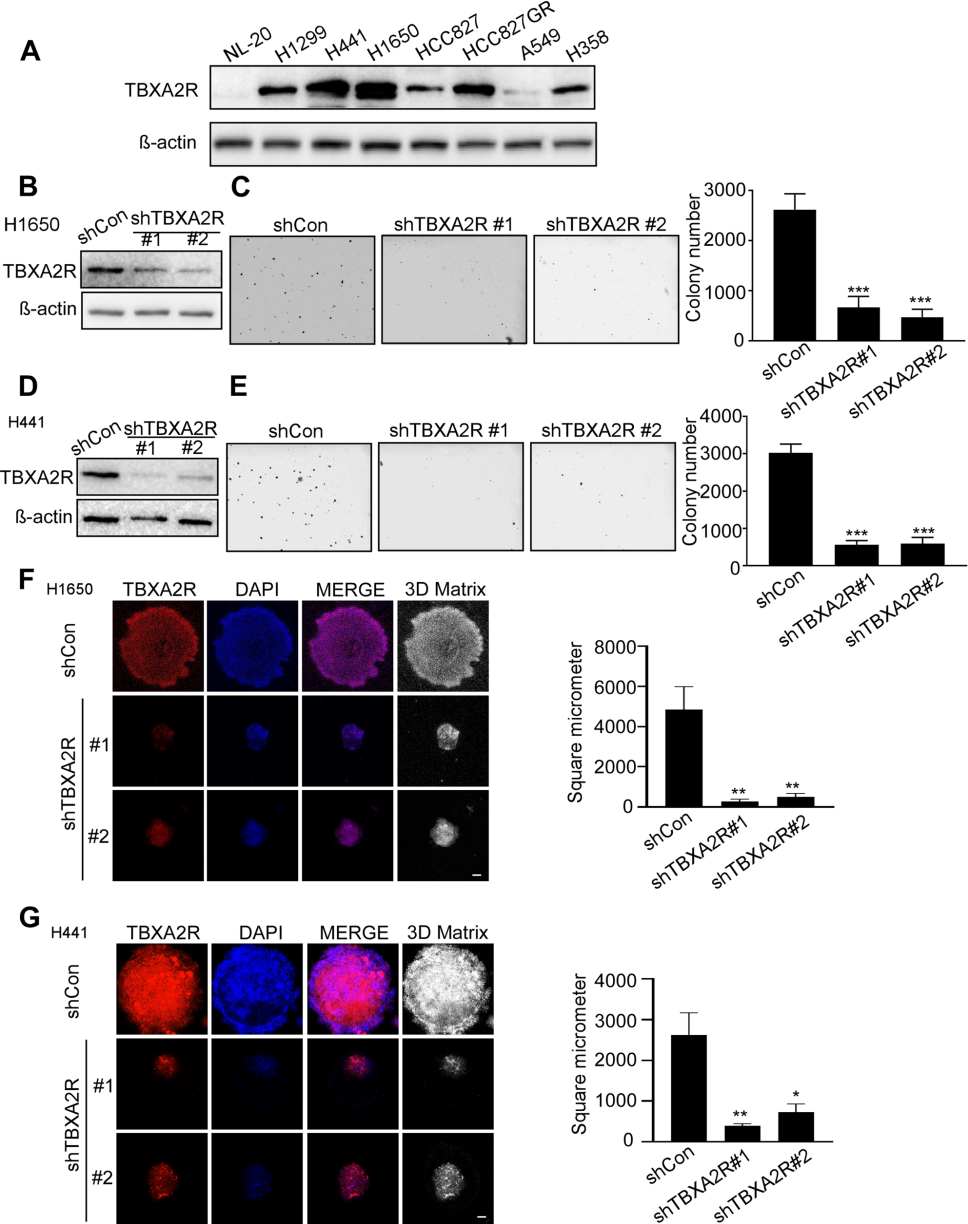
TBXA2R knockdown inhibits the proliferation of H1650 and H441 lung cancer cell lines. **A**, comparative analysis of TBXA2R expression levels in normal human lung cells versus multiple lung cancer cell lines. **B**, establishment of stable TBXA2R knockdown in H1650 lung cancer cells, confirmed by Western blot analysis. **C**, assessment of the effect of TBXA2R knockdown on the anchorage-independent growth of H1650 cells. **D**, generation of H441 lung cancer cells with stable TBXA2R knockdown. **E**, analysis of TBXA2R knockdown impact on the anchorage-independent growth of H441 cells. Cells stably expressing either non-targeting shRNA (shNT) or TBXA2R-targeting shRNA (shTBXA2R) were cultured in 1.25% agar, and colony formation was quantified using microscopy and Image-Pro Plus software (version 6). Results are presented as mean values ± SD from 3 independent experiments. Statistical significance was determined using the Student’s t-test, with asterisks indicating significant differences between TBXA2R knockdown and control groups. **F**,** G**, immunofluorescence analysis of 3D multicellular spheroids derived from H1650 and H441 cells. TBXA2R is indicated in red, with nuclei counterstained with DAPI (blue). Scale bar represents 10 μm (*, *p* < 0.05; **, *p* < 0.01; ***, *p* < 0.001, one-way ANOVA)

We evaluated the protein expression levels of TBXA2R across various lung cancer cell lines. Our findings indicated that TBXA2R expression is elevated in human non-small cell lung cancer (NSCLC) cell lines compared with normal NL-20 lung cells. Among these, the adenocarcinoma cell lines H1650 and H441, selected for further investigation, exhibit the highest TBXA2R expression levels (Fig. 2A). TBXA2R knockdown in H1650 cells was achieved using shTBXA2R (Fig. 2B), resulting in a reduction in colony formation as assessed by an anchorage-independent cell growth assay (Fig. 2C). Additionally, the knockdown of TBXA2R also inhibits the growth of H441 cells (Fig. 2D and E). To further elucidate the role of TBXA2R in lung cancer cell growth, we conducted an analysis of 3-D multicellular spheroids using immunofluorescence staining. The results demonstrated that TBXA2R knockdown significantly impairs the growth of both H1650 (Fig. 2F) and H441 (Fig. 2G) cells in a 3-D culture environment. These results suggest that inhibiting TBXA2R expression markedly diminishes the independent growth capability of these lung cancer cells.

### TBXA2R suppresses lung cancer cell growth through modulation of the PI3K-AKT and cytokine-cytokine receptor interaction pathways

**Fig. 3:**
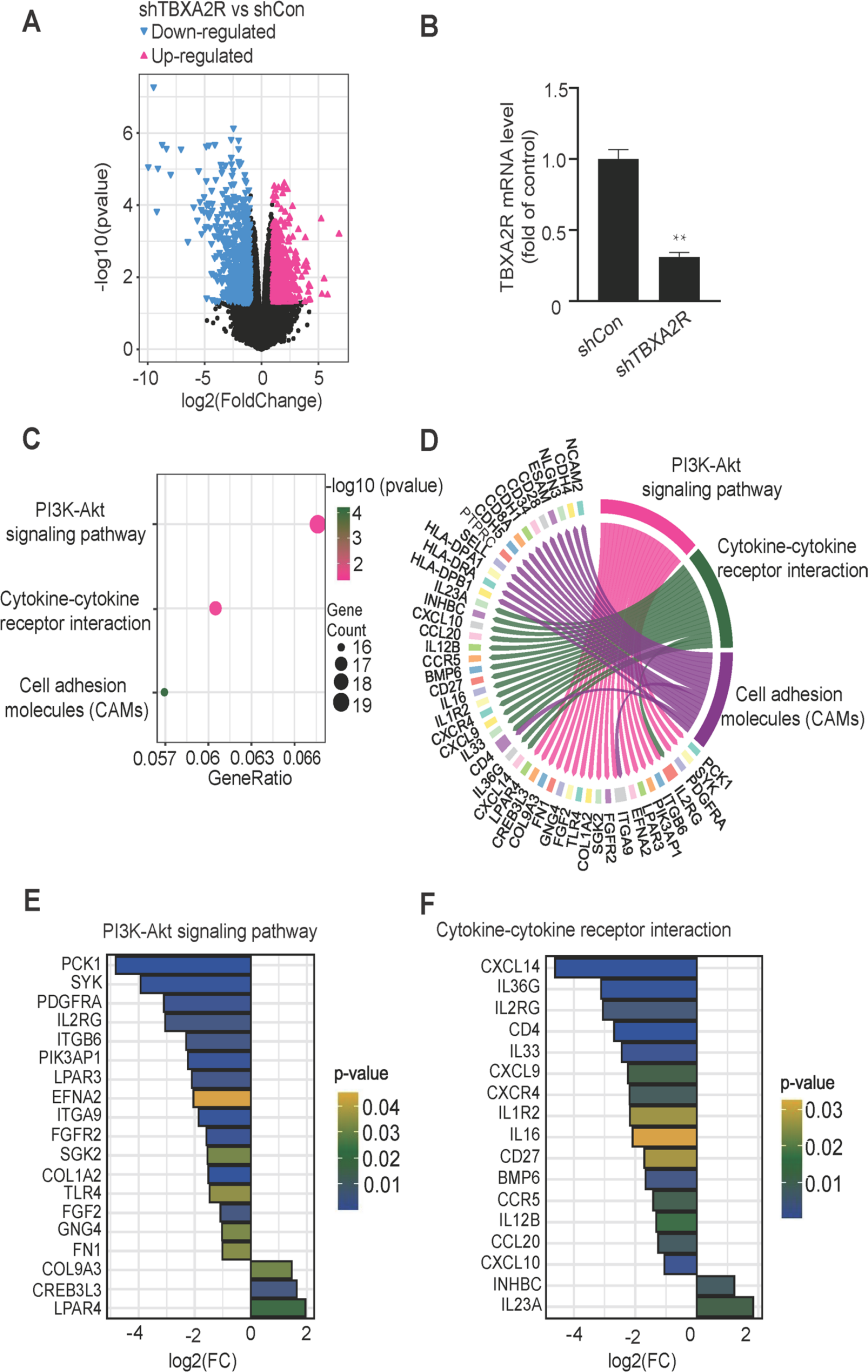
RNA-seq analysis of the gene changes in response to TBXA2R knockdown (shTBXA2R) in H1650 cells. **A**, comparison of TBXA2R expression levels between shTBXA2R and WT (shCon) as determined by RNA-seq data. **B**, volcano plot depicting gene expression changes (shTBXA2R vs. shCon; fold change ≥ 2; *p* value < 0.05). **C**, KEGG analysis of differentially expressed genes presented in a bubble chart. **D**, cluster plot showcasing the expression profiles of genes associated with the identified signaling pathways. **E**, fold changes and *p* values of genes in the PI3K-AKT pathway. **F**, fold changes and *p* values of genes in the cytokine-cytokine receptor interaction pathway

To explore the mechanisms by which TBXA2R functions in lung adenocarcinoma, RNA sequencing (RNAseq) was conducted on shCon and shTBXA2R H1650 cells. The knockdown of TBXA2R led to a significant reduction in its mRNA levels (Fig. 3A). Volcano plots were generated to identify differentially expressed genes, revealing 619 upregulated genes and 747 downregulated genes (≥ 2-fold difference, *p* < 0.05, Fig. 3B). Subsequent Kyoto Encyclopedia of Genes and Genomes (KEGG) pathway analysis indicates enrichment of the PI3K-AKT, cytokine-cytokine receptor interaction, and cell adhesion molecules (CAMs) pathways (*p* < 0.05). The cluster plot from the KEGG enrichment analysis depicts the expression spectrum of genes involved in these pathways (Fig. 3C, D). Additionally, fold changes and p-values for genes in the PI3K-AKT and cytokine-cytokine receptor interaction pathways are provided (Fig. 3E, F).

**Fig. 4:**
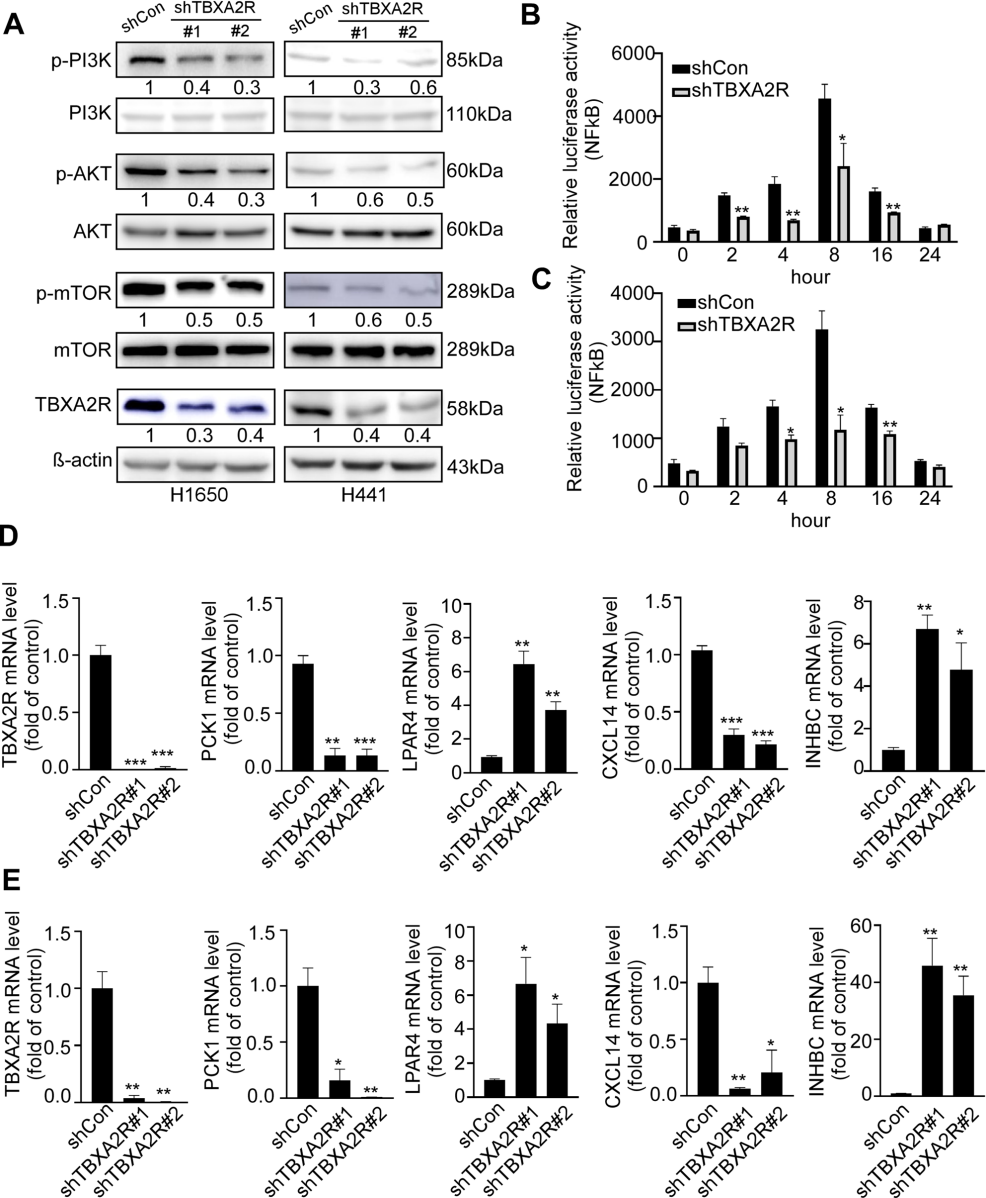
Effects of TBXA2R knockdown on the PI3K/AKT/mTOR signaling pathway and NF-κB activation in lung cancer cells. **A**, TBXA2R knockdown results in decreased phosphorylation levels within the PI3K/AKT/mTOR signaling pathway. **B**,** C**, H1650 and H441 cells with TBXA2R knockdown (shTBXA2R) and control (shCon) were treated with urethane for specified durations; subsequent NF-κB nuclear translocation was evaluated using a luciferase assay. **D**,** E**, differential expression of key genes in the PI3K-AKT pathway (PCK1, LPAR4) and the cytokine-cytokine receptor interaction pathway (CXCL4, INHBC) was confirmed through RT-PCR analysis. Data are presented as means ± SD from triplicate experiments conducted independently 3 times (*, *p* < 0.05; **, *p* < 0.01, one-way ANOVA)

To investigate TBXA2R’s impact on kinase activation within the PI3K-AKT pathway, we observed that TBXA2R knockdown significantly reduces the phosphorylation levels of PI3K, AKT, and mTOR, while total protein levels remained unchanged in both H1650 and H441 cells (Fig. 4A). NF-κB, a crucial transcription factor regulated by the PI3K-AKT pathway and significant in lung cancer development, was also found to have its transcriptional activation inhibited by TBXA2R knockdown, as demonstrated by a luciferase assay (Fig. 4B, C). Further validation of differentially expressed genes from the PI3K-AKT and cytokine-cytokine receptor interaction pathways (Fig. 3E, F) revealed that TBXA2R knockdown significantly decreases PCK1 and CXCL14 expression while increasing LPAR4 and INHBC expression in both H1650 (Fig. 4D) and H441 (Fig. 4E) cells. In summary, our findings indicate that the knockdown of TBXA2R effectively suppresses lung cancer cell growth primarily through the modulation of the PI3K-AKT and cytokine-cytokine receptor interaction pathways.

### Knockdown of TBXA2R suppresses EGFR expression by inhibiting NF-κB p52 nuclear translocation in lung cancer cells

**Fig. 5:**
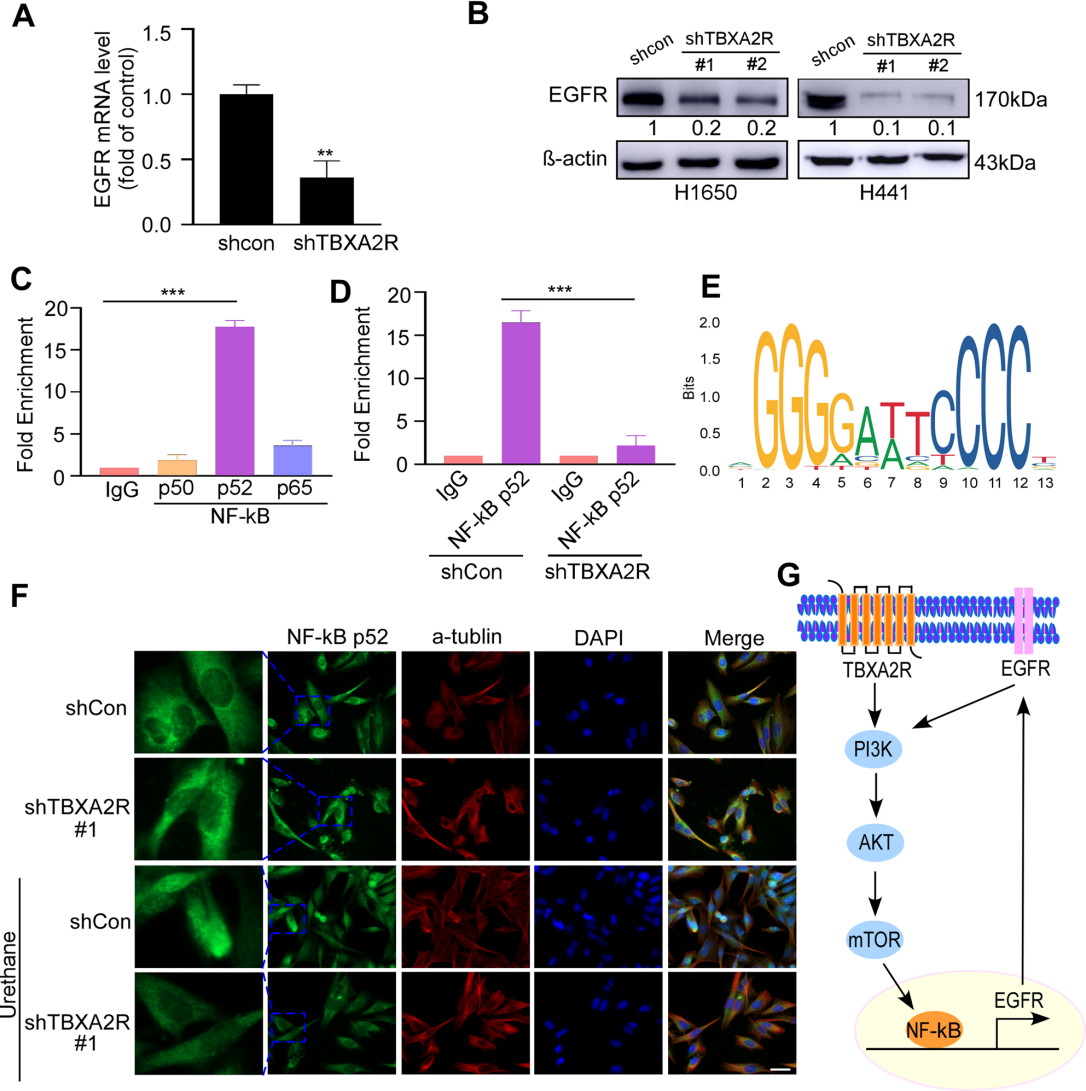
Knocking down TBXA2R expression suppresses EGFR by inhibiting NF-κB p52 nuclear translocation in lung cancer cells. **A**, RNA-seq analysis comparing EGFR expression levels between TBXA2R knockdown (shTBXA2R) and wild-type (shCon) cells. **B**, Western blot analysis illustrating the impact of TBXA2R knockdown on EGFR protein levels in H1650 and H441 cell lines. **C**, ChIP-qPCR showing the interaction of the NF-κB protein with the *EGFR* gene promoter. **D**, knockdown of TBXA2R leads to a decrease in EGFR expression by modulating its promoter activity through NF-κB p52. **E**, predicted binding motif for NF-κB p52 within the *EGFR* promoter region. **F**, immunofluorescence analysis of H1650 cells with stable TBXA2R knockdown, treated with urethane for 24 h, assessing NF-κB p52 localization (scale bars represent 50 μm). Data are presented as means ± SD from 3 independent experiments conducted in triplicate (*, *p* < 0.05; **, *p* < 0.01, one-way ANOVA)

EGFR is critical in the pathogenesis of non-small cell lung cancer (NSCLC), and targeted therapies against EGFR have notably improved patient prognosis, particularly for those with EGFR mutations [16–18, 28, 29]. Through RNA sequencing analysis **(**Fig. 3), we found that silencing TBXA2R results in a marked decrease in *EGFR* mRNA expression (Fig. 5A). To support this finding, we further demonstrated that TBXA2R knockdown also substantially lowers EGFR protein levels in the H1650 and H441 NSCLC cell lines (Fig. 5B). This suggests a potential regulatory mechanism involving NF-κB. Chromatin immunoprecipitation with quantitative PCR (ChIP-qPCR) analysis confirmed that NF-κB p52 directly binds to the *EGFR* promoter to regulate its expression, while other NF-κB subunits like p50 and p65 showed no detectable binding (Fig. 5C). Importantly, shTBXA2R knockdown significantly reduces the binding of NF-κB p52 to the *EGFR* promoter, thereby modulating EGFR expression (Fig. 5D). Using motif prediction tools from JASPAR (https://jaspar2022.genereg.net/downloads/), we identified a specific binding site for NF-κB p52 on the *EGFR* promoter (Fig. 5E). Furthermore, we found that TBXA2R knockdown impairs NF-κB p52 translocation to the nucleus in H1650 cells (Fig. 5F), and similar results were observed in H441 cells (Supplementary Fig. 1). Collectively, these results show that TBXA2R regulates EGFR expression through the PI3K/AKT/mTOR/NF-κB signaling axis (Fig. 5G).

### Loss of TBXA2R decelerates tumor growth in urethane-induced lung carcinogenesis

**Fig. 6:**
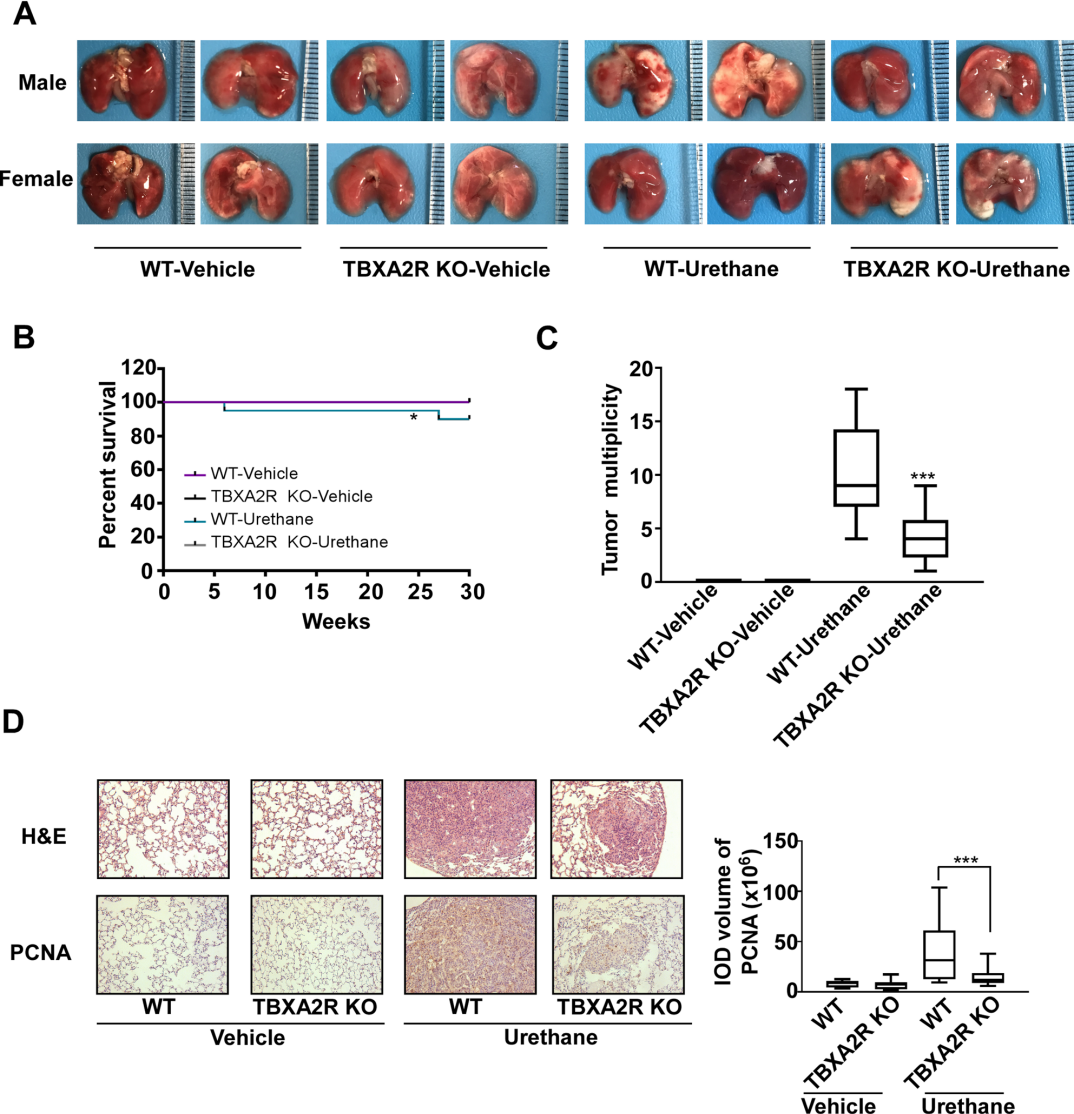
Loss of TBXA2R inhibits urethane-induced lung carcinogenesis. **A**, WT and TBXA2R KO mice were used in the study. Mice received intraperitoneal injections of urethane (1 g/kg in PBS) or vehicle (PBS) once a week for 10 weeks. Lungs were collected 30 weeks following the initial urethane administration. **B**, body weight comparison across different groups. **C**, tumor multiplicity analysis revealed an average of 4.2 ± 2.1 tumors in urethane-treated TBXA2R KO mice compared to 10.1 ± 4.1 tumors in the urethane-treated WT group. No significant sex differences in tumor numbers were observed between male and female mice. **D**, lung tissue samples were harvested and stained with hematoxylin and eosin (H&E). Immunohistochemical analysis was performed to assess the expression of proliferating cell nuclear antigen (PCNA) in the lungs of urethane-treated mice versus vehicle-treated controls. Data are presented as means ± SD (*, *p* < 0.05; **, *p* < 0.01, one-way ANOVA)

Our research into the functional implications of TBXA2R in lung tumorigenesis led us to propose that TBXA2R-deficient mice would show a different susceptibility to chemically induced lung tumors compared to wild-type (WT) mice. To investigate this hypothesis, we utilized a urethane-induced lung carcinogenesis mouse model [30, 31]. Urethane, a recognized carcinogen present in tobacco leaves and smoke, is frequently used to mimic human lung adenocarcinoma. In our study, WT and TBXA2R KO mice received weekly intraperitoneal injections of 1 g/kg urethane for 10 consecutive weeks, while control mice were administered a vehicle (1× PBS, i.p.) during the same timeframe. Tumor counts were conducted at 30 weeks post-treatment. The results revealed that TBXA2R KO mice developed significantly fewer lung tumors than their WT counterparts (Fig. 6A; SupplementaryFig. S2A). Importantly, WT mice treated with urethane exhibited signs of illness and a lower survival rate compared with the control group (*p* < 0.05; Fig. 6B). Specifically, TBXA2R KO mice had an average of 4.2 ± 2.1 tumors, while WT mice averaged 10.1 ± 4.1 tumors (***, *p* < 0.001; Fig. 6C). Histological analysis through H&E staining demonstrate that tumors in WT mice disrupted the normal alveolar architecture, characterized by an increased nuclear/cytoplasmic ratio and cytological atypia typical of adenomas. In contrast, TBXA2R KO mice display fewer adenomas, with their lung tissue largely preserving normal alveolar structure (Fig. 6D). Additionally, the expression of proliferating cell nuclear antigen (PCNA), a marker for cell proliferation, was significantly diminished in tumor tissues from TBXA2R KO mice compared to WT mice (Fig. 6D). Notably, no significant differences in body weight were observed among the various groups (Supplementary Fig. 2B). Conversely, TBXA2R KO mice treated with urethane showed a markedly higher survival rate than the control group. Genotyping of all mice was confirmed through PCR analysis of lung tissue (Supplementary Fig. 2C).

In summary, our findings indicate that the lack of TBXA2R signaling correlates with a reduction in tumor growth during urethane-induced lung carcinogenesis.

## Discussion

This study highlights the viability of TBXA2R as a novel therapeutic target as a novel therapeutic target in NSCLC progression. Although significant strides have been made in NSCLC treatment through targeted therapies and immunotherapies, challenges such as therapeutic resistance and limited efficacy in specific patient populations persist [32, 33]. While current treatments, including EGFR inhibitors, have contributed to improved patient outcomes, resistance mechanisms frequently limit their effectiveness [32, 34], thereby necessitating the search for alternative targets.

A major finding in this study is the inverse correlation between TBXA2R expression and patient survival, suggesting a role in disease aggressiveness (Fig. 1). Functionally, the knockout of TBXA2R resulted in marked inhibition of NSCLC cell proliferation (Fig. 2), suggesting a pro-tumorigenic function for TBXA2R. Furthermore, TBXA2R was shown to activate multiple signaling pathways, notably enhancing PI3K-AKT signaling, which is a well-established driver of NSCLC progression [35–37]. The elevated TBXA2R levels in the EGFR inhibitor-resistant HCC827GR cell line compared to its sensitive counterpart highlight a potential mechanism by which TBXA2R could play a mechanistic role in resistance (Fig. 2A). This finding suggests that co-targeting TBXA2R and EGFR may have implications for combination therapies, which should be further investigated.

Furthermore, the crosstalk between TBXA2R and EGFR signaling may reveal previously unrecognized mechanisms contributing to NSCLC progression. Given the central role of EGFR inhibitors in NSCLC therapy, understanding how TBXA2R modulates this pathway could pave the way for more effective combination treatment strategies. Simultaneous targeting of TBXA2R and EGFR may not only enhance therapeutic efficacy but also help overcome resistance, a common challenge in EGFR-targeted therapies. In addition, the NF-κB signaling pathway, another key regulator of NSCLC, is significantly influenced by TBXA2R activation [38]. NF-κB governs the expression of genes involved in inflammation, immune response, and cell survival—each crucial for cancer development and progression [24]. Interestingly, NF-κB also regulates EGFR expression, creating a positive feedback loop that amplifies oncogenic signaling (Fig. 5). This interconnection between NF-κB and EGFR, mediated by TBXA2R, highlights the complexity of NSCLC-associated signaling networks. Disrupting this loop could provide a novel therapeutic approach for attenuating aggressive NSCLC phenotypes. Prior studies support this hypothesis, showing that NF-κB enhances EGFR expression, and that knocking down IKKα or IKKβ can significantly reduce EGFR mRNA and protein levels [25, 39]. Moreover, NF-κB can interact with Bcl-3, which directly binds to the *EGFR* promoter, further driving EGFR expression [40]. This dynamic interaction between NF-κB and EGFR contributes to the aggressive nature of NSCLC cells, perpetuating tumor growth and therapy resistance. Targeting TBXA2R may influence this feedback loop, potentially affecting NF-κB activity and EGFR expression, warranting further experimental validation.

TBXA2R functions as a G-protein–coupled receptor that activates downstream signaling through G protein subunits such as G12/13 and Gαq/11. These intermediates directly engage PI3K, resulting in phosphorylation and activation of AKT to promote tumor cell survival and proliferation [41, 42]. In parallel, TBXA2R signaling can stimulate PKC and other kinases that converge on NF-κB, driving pro-survival and pro-inflammatory transcriptional programs [43, 44]. Importantly, this mode of regulation is distinct from that of receptor tyrosine kinases such as EGFR, which activate PI3K primarily through adaptor proteins such as GAB1 and GAB2 [45]. By coupling extracellular thromboxane A2 to intracellular PI3K-AKT and NF-κB pathways via G-proteins, TBXA2R establishes a parallel signaling axis that can complement or bypass EGFR activity. This mechanistic diversity highlights TBXA2R’s unique upstream position and underscores its potential as a novel therapeutic target in NSCLC.

Our in vivo findings suggest that TBXA2R may contribute to lung tumorigenesis, as its deletion led to a significant reduction in urethane-induced lung tumor development in a mouse model [30, 31, 46]. These results indicate a potential relevance of TBXA2R in NSCLC and suggest that it may also be involved in broader aspects of lung carcinogenesis, which requires further validation. By diminishing tumor burden and lung injury, TBXA2R knockout provides a strong rationale for exploring its inhibition as a preventive strategy in lung cancer models.

Our findings suggest that TBXA2R inhibition could be integrated into current NSCLC treatment strategies to enhance therapeutic efficacy and overcome resistance. TBXA2R signaling activates the PI3K-AKT and NF-κB pathways, which are frequently implicated in resistance to EGFR-targeted therapies [47, 48]. Thus, combining TBXA2R inhibition with EGFR inhibitors, such as Osimertinib, may suppress bypass signaling and restore drug sensitivity. Previous reports indicate that GPCR-driven activation of PI3K-AKT can sustain tumor growth despite EGFR blockade [49]. Furthermore, because NF-κB activation contributes to an immunosuppressive microenvironment [50, 51], TBXA2R inhibition may also complement immune checkpoint blockade with anti–PD-1/PD-L1 therapy. In addition, chemotherapy-induced stress responses often converge on GPCR or PI3K signaling [52], raising the possibility that TBXA2R inhibition could have additive or synergistic effects when combined with conventional cytotoxic regimens. Together, these considerations highlight the translational potential of TBXA2R-targeted therapies both in overcoming resistance and in optimizing combination strategies in NSCLC.

Despite these promising implications, several limitations of this study should be noted. Although our in vitro models provide mechanistic insight, further validation in patient-derived xenografts or resistant models will be necessary. The lack of clinically validated TBXA2R-specific inhibitors also presents a translational challenge, as the development of selective small molecules or antibodies remains in early stages. Finally, while we propose the integration of TBXA2R inhibition with current therapeutic strategies, future studies will be required to confirm the potential of TBXA2R targeting.

In conclusion, our study suggests that TBXA2R may play an important role in the pathophysiology of NSCLC, being associated with key signaling pathways that contribute to tumor progression and therapy resistance. These findings indicate that TBXA2R could serve as a potential therapeutic target, warranting further investigation. Additional studies to elucidate TBXA2R’s mechanisms and interactions may provide valuable insights that inform the development of novel strategies for managing this challenging disease.

## Supplementary Information

Below is the link to the electronic supplementary material.

Supplementary Material 1

Supplementary Material 2

Supplementary Material 3

Supplementary Material 4

Supplementary Material 5

## Data Availability

No datasets were generated or analysed during the current study.
