# Supplementary material for: The mechanistic role of the thromboxane A2 receptor (TBXA2R) in non-small cell lung cancer (NSCLC)
Source: Cancer Cell Int. 2026 Apr 28;26:231. doi: 10.1186/s12935-026-04283-6 (PMC13270695; doi:10.1186/s12935-026-04283-6)
Supplement: Supplementary file 1 — Supplementary Material 1 [file 12935_2026_4283_MOESM1_ESM.docx]

**Figure S1. Knockdown of TBXA2R inhibit NFκB p52 nuclear translocation in H441 cells.** H441 cells with stable knockdown of TBXA2R were treated with urethane for 24h and then the localization of NFκB p52 was determined by immunofluorescence analysis (scale bars, 50μm).

**Figure S2. Loss of TBXA2R suppresses urethane-induced lung carcinogenesis.** **A,** Lung tissues from WT and TBXA2R KO mice. **B,** body weight comparison between groups. **C,** Genotyping of lung tissues from mice was performed using standard PCR analysis following the Jackson Laboratory genotyping protocol.
