# Supplementary material for: The mechanistic role of the thromboxane A2 receptor (TBXA2R) in non-small cell lung cancer (NSCLC)
Source: Cancer Cell Int. 2026 Apr 28;26:231. doi: 10.1186/s12935-026-04283-6 (PMC13270695; doi:10.1186/s12935-026-04283-6)
Supplement: Supplementary file 2 — Supplementary Material 2 [file 12935_2026_4283_MOESM2_ESM.docx]

**Bioinformatics analysis**

*Sequence and filtering of clean reads*

A cDNA library was constructed by using pooled RNA from mouse skin samples and sequencing was performed by using the Illumina NovaseqTM 6000 sequence platform. Using the Illumina paired-end RNA-seq approach, we sequenced the transcriptome, generating a total of 2 million x 150 bp paired-end reads. Reads obtained from the sequencing analysis included adapters or low-quality bases that may have the potential to affect the assembly and analysis. Thus, to obtain high quality clean reads, the reads were further filtered by Cutadapt (<https://cutadapt.readthedocs.io/en/stable/,version:cutadapt-1.9> (1). The parameters were as follows: 1) removing reads containing adapters; 2) removing reads containing polyA and polyG; 3) removing reads containing more than 5% of unknown nucleotides (N); 4) removing low quality reads containing more than 20% of low quality (Q-value ≤20) bases ([http://www.bioinformatics.babraham.ac.uk/projects/fastqc/,0.11.9)](http://www.bioinformatics.babraham.ac.uk/projects/fastqc/,0.11.9)%20%7bThompson,%202020#38}) , including the Q20, Q30 and GC-content of the clean data. After that, a total of G bp of cleaned, paired-end reads were produced.

*Alignment with reference genome*

We aligned reads of all samples to the < research species > reference genome using the HISAT2 (<https://daehwankimlab.github.io/hisat2/,version:hisat2-2.0.4>) package, which initially removes a portion of the reads based on quality information accompanying each read and then maps the reads to the reference genome. HISAT2 allows multiple alignments per read (up to 20 by default) and a maximum of two mismatches when mapping the reads to the reference. HISAT2 builds a database of potential splice junctions and confirms these by comparing the previously unmapped reads against the database of putative junctions (2-4).

*Quantification of gene abundance*

The mapped reads of each sample were assembled by using StringTie (<http://ccb.jhu.edu/software/stringtie/,version:stringtie-1.3.4d>) with default parameters. Then, all transcriptomes from all samples were merged to reconstruct a comprehensive transcriptome using gffcompare software (<http://ccb.jhu.edu/software/stringtie/gffcompare.shtml,version:gffcompare-0.9.8>). After the final transcriptome was generated, StringTie and ballgown (http://www.bioconductor.org/packages/release/bioc/html/ballgown.html) were used to estimate the expression levels of all transcripts and perform expression abundance for mRNAs by calculating FPKM (fragment per kilobase of transcript per million mapped reads) value (4-6).

**References**

1. Martin M. Cutadapt removes adapter sequences from high-throughput sequencing reads. EMBnet journal **2011**;17:10-2

2. Kim D, Paggi JM, Park C, Bennett C, Salzberg SL. Graph-based genome alignment and genotyping with HISAT2 and HISAT-genotype. Nature biotechnology **2019**;37:907-15

3. Kim D, Langmead B, Salzberg SL. HISAT: a fast spliced aligner with low memory requirements. Nature methods **2015**;12:357-60

4. Pertea M, Kim D, Pertea GM, Leek JT, Salzberg SL. Transcript-level expression analysis of RNA-seq experiments with HISAT, StringTie and Ballgown. Nature protocols **2016**;11:1650-67

5. Kovaka S, Zimin AV, Pertea GM, Razaghi R, Salzberg SL, Pertea M. Transcriptome assembly from long-read RNA-seq alignments with StringTie2. Genome biology **2019**;20:1-13

6. Pertea M, Pertea GM, Antonescu CM, Chang T-C, Mendell JT, Salzberg SL. StringTie enables improved reconstruction of a transcriptome from RNA-seq reads. Nature biotechnology **2015**;33:290-5
