# Supplementary material for: The mechanistic role of the thromboxane A2 receptor (TBXA2R) in non-small cell lung cancer (NSCLC)
Source: Cancer Cell Int. 2026 Apr 28;26:231. doi: 10.1186/s12935-026-04283-6 (PMC13270695; doi:10.1186/s12935-026-04283-6)
Supplement: Supplementary file 3 — Supplementary Material 3 [file 12935_2026_4283_MOESM3_ESM.docx]

| Gene name | Forward sequence | Reverse sequence |
| --- | --- | --- |
| TBXA2R | CCTGGGTGTATATCCTGTTCCG | AGATCGTGCCACTGTACTCCAG |
| PCK1 | CATTGCCTGGATGAAGTTTGACG | GGGTTGGTCTTCACTGAAGTCC |
| LPAR4 | CCACCTGCTTTGAAGGCTTCTC | AGGCTTGCGAAGAGTTCTCAGC |
| CXCL14 | ACCCA TTTTGTGTGGAGGAGA | TGCTCACCTCTCACTTTCGT3 |
| INHBC | CTGTGTCCAGAGCTGCTTTGAG | AGACGAGTCTGGTTGATGGTGG |
| EGFR | CATTATCCGACGCTGGCTCT | GGAGCAGAGGAGGAGGAGAA |
| GAPDH | CTCTGCTCCTCCTGTTCGAC3 | GCCCAATACGACCAAATCC |

Supplementary Table 1. The primer sequences.
