# Supplementary figures and images for: The mechanistic role of the thromboxane A2 receptor (TBXA2R) in non-small cell lung cancer (NSCLC)
Source: Cancer Cell Int. 2026 Apr 28;26:231. doi: 10.1186/s12935-026-04283-6 (PMC13270695; doi:10.1186/s12935-026-04283-6)

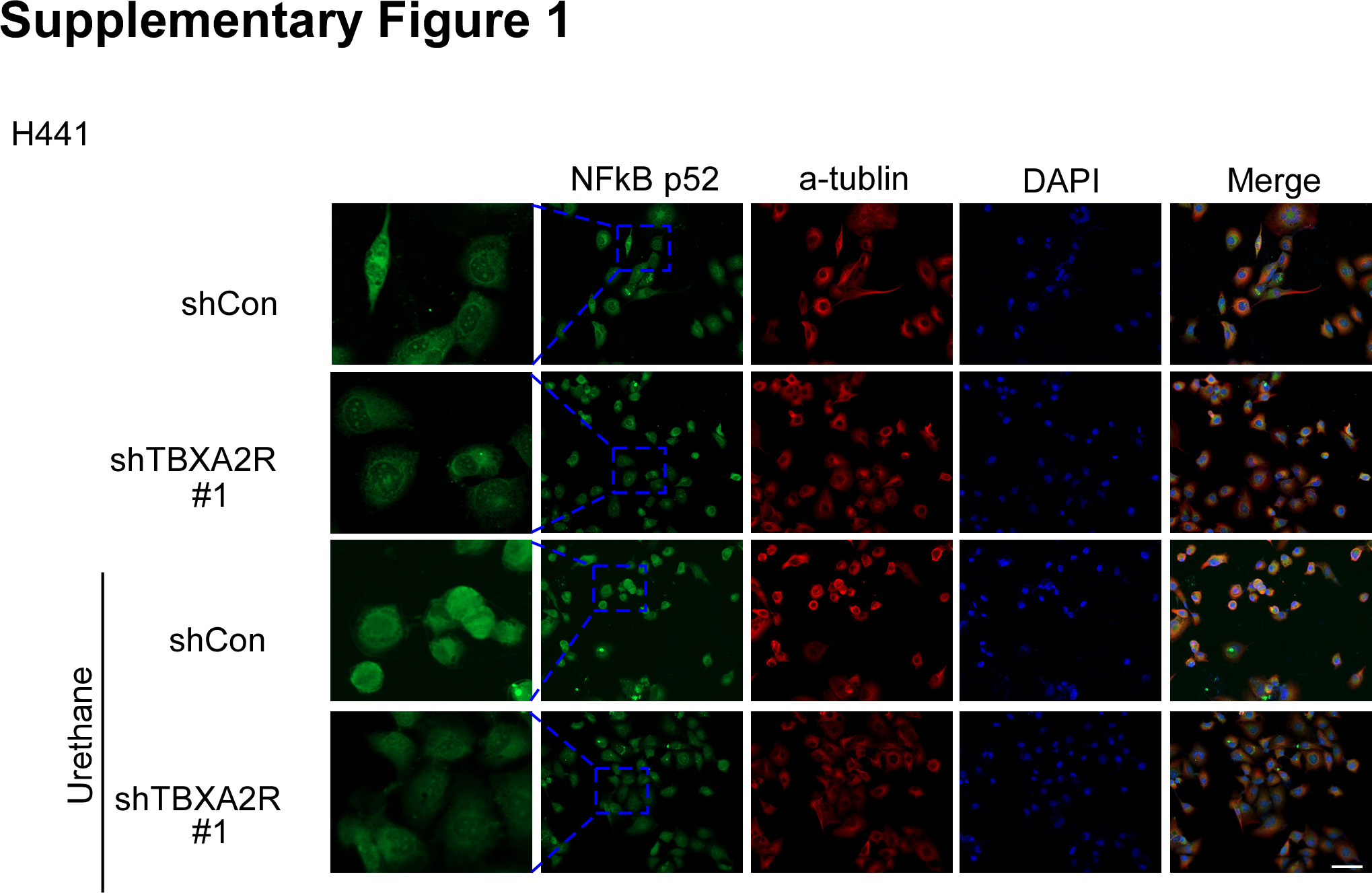

Supplement: Supplementary file 4 — Supplementary Material 4 [file 12935_2026_4283_MOESM4_ESM.tif]

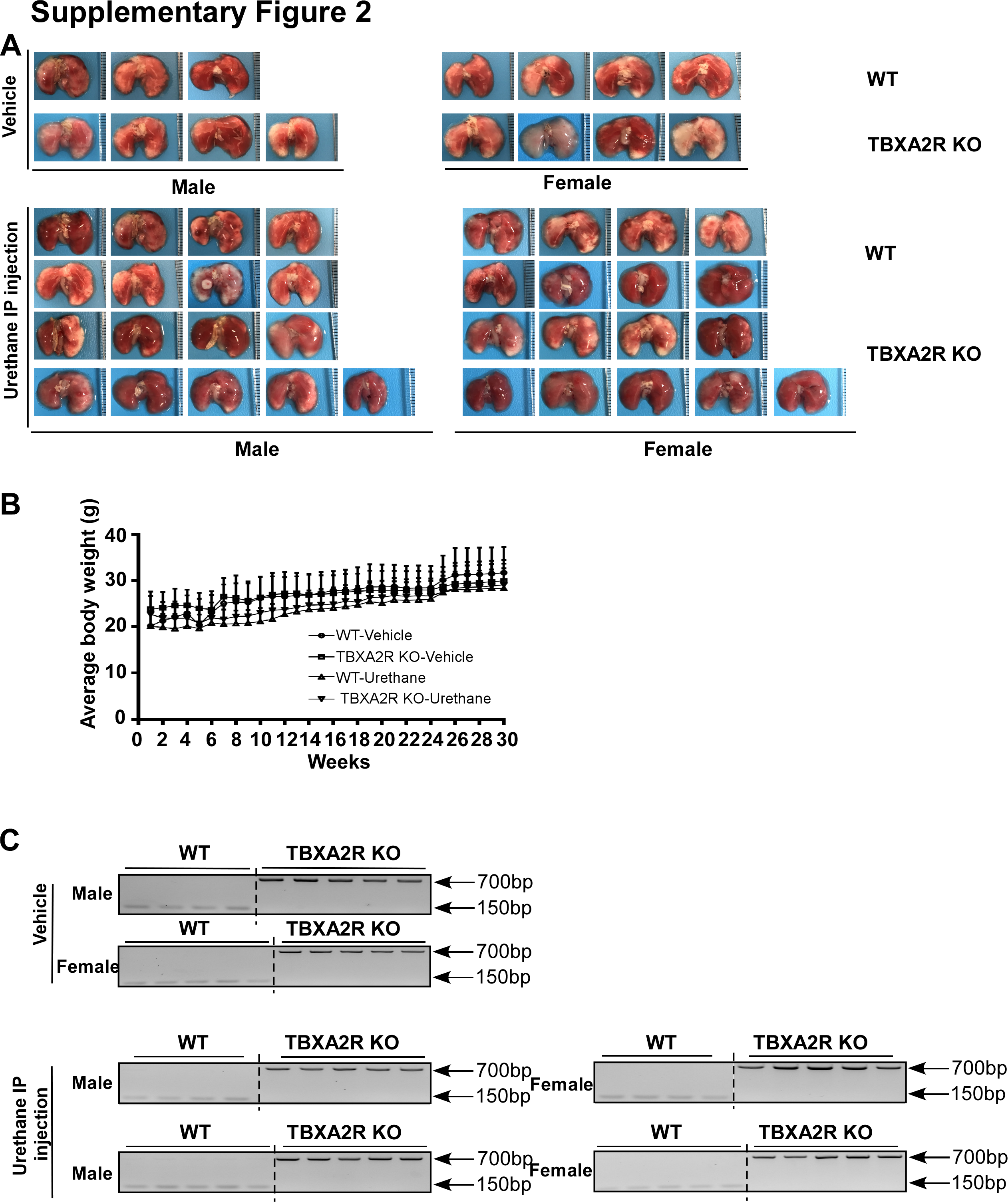

Supplement: Supplementary file 5 — Supplementary Material 5 [file 12935_2026_4283_MOESM5_ESM.tif]
